# Synthesis, Characterization, and Anticancer Activity of New Metal Complexes Derived from 2-Hydroxy-3-(hydroxyimino)-4-oxopentan-2-ylidene)benzohydrazide

**DOI:** 10.1155/2015/126023

**Published:** 2015-06-25

**Authors:** Abdou Saad El-Tabl, Moshira Mohamed Abd El-Waheed, Mohammed Ahmed Wahba, Nahla Abd El-Halim Abou El-Fadl

**Affiliations:** ^1^Department of Chemistry, Faculty of Science, Menoufia University, P.O. Box 5744, Shibin Al Kawm, Egypt; ^2^Department of Pathology, Faculty of Medicine, Menoufia University, P.O. Box 5744, Shibin Al Kawm, Egypt; ^3^Inorganic Chemistry Department, National Research Centre, Dokki, Cairo 12311, Egypt

## Abstract

Novel metal(II) complexes derived from 2-hydroxy-N′-((Z)-3-(hydroxyimino)-4-oxopentan-2-ylidene)benzohydrazide ligand (H_2_L) were synthesized and characterized by elemental and thermal analyses (DTA and TGA), IR, UV-VIS, ^1^H-NMR, ESR and mass spectroscopy, magnetic susceptibilities, and conductivities measurements. The complexes adopt distorted octahedral geometry. The ESR spectra of the solid copper(II) complexes are characteristic to d^9^ configuration and have an axial symmetry type of a d_(*x*^2^-*y*^2^)_ ground state. The *g* values confirmed the tetragonal octahedral geometry with a considerably ionic or covalent environment. The cytotoxic activity of the ligand and its metal complexes showed potent cytotoxicity effect against growth of human liver cancer HepG2 cell lines compared to the clinically used Sorafenib (Nexavar).

## 1. Introduction

There is a growing interest in oxime-hydrazone and their coordination compounds caused by their biological activity [1, 2]. Many clinically successful anticancer drugs were either naturally occurring molecules or have been developed from their synthetic analogs. Metal complexes have unique properties enhancing their role as antitumor agents. An important property is the ability of metals to form positively charged ions in an aqueous solution that can bind to negatively charged biological molecules [3, 4]. The high electron affinity of metal ions can significantly polarize groups that are coordinated to them, leading to the generation of hydrolysis reactions [4]. Furthermore, metal ions also has the ability to coordinate ligands in a three dimensional configuration, thus allowing functionalization of groups that can be tailored to defined molecular targets [5, 6]. Recently, considerable attention has been drawn to oximes, hydrazones, and their coordinated compounds due to their biological activities as fungicides [7, 8], bactericides [9], analgesic and anti-inflammatory [10], antioxidant [11, 12], antitumor [13–15], and insecticidal [16]. Metal complexes of bis-hydrazone derived from isatin monohydrazone and 2-hydroxy-l-naphthaldehyde have been reported and they demonstrated interesting biological properties [17]. Oxovanadium(IV) complexes derived from 2-thiophene carboxylic acid hydrazide showed a great cytotoxicity towards Artemia salina [18]. Homo- and heteronuclear copper(II) and nickel(II) complexes derived from oxime-type ligands have been also reported; the observed IC_50_ values indicated that they are potential antioxidant [19]. Cytotoxicity of a series of cobalt(II) complexes of 2-furaldehyde oximes was compared with copper complexes of furan oximes to determine whether the type of metal is important to the cytotoxicity and mode of action of the complexes. It was shown that varying the type of metal produces differences in both cytotoxicity and mode of action [20]. Oxime hydrazones can react with metal(II) salts to produce either mono- or binuclear complexes. The keto hydrazone moiety may coordinate to metals in the ketoamide or deprotonated enolimine form. Compounds containing both oxime and hydrazone groups are typically act as tridentate, mono- or biprotic ligands coordinating through the amide oxygen, imine, and oxime nitrogen atom depending on the reaction conditions [21]. Considerable interest has been attracted to synthesize oxime-hydrazide compounds as important target structures and evaluated their biological activities. These observations have been guiding the development of new compounds that possess varied biological activities. In view of interest and importance of oxime-hydrazide complexes, we reported here synthesis and characterization of new metal complexes derived from 2-hydroxy-N′-((Z)-3-(hydroxyimino)-4-oxopentan-2-ylidene)benzohydrazide. The work was extended to study the anticancer activity of the ligand and its metal complexes against human liver cancer HepG2.

## 2. Experimental

### 2.1. Instrumentation

All reagents employed for the preparation of the ligand and its complexes were of the analytical grade available and used without further purification. Metal salts and salicylic hydrazide were provided from SIGMA-ALDRICH company; diacetylmonoxime was prepared by a published method [22]. The purity of all compounds was confirmed by TLC. The ligand and its metal complexes were analyzed for C, H, and N at the Microanalytical center, Cairo University, Egypt. Standard analytical methods were used to determine the metal ion content [23]. FT-IR spectra of the ligand and its metal complexes were measured using KBr discs by a Jasco FT/IR 300E Fourier transform infrared spectrophotometer covering the range 400–4000 cm^−1^. Electronic spectra in the 200–900 nm regions were recorded on a Perkin-Elmer 550 spectrophotometer. The thermal analyses (DTA and TGA) were carried out on a Shimadzu DT-30 thermal analyzer from room temperature to 800°C at a heating rate of 10°C/min. Magnetic susceptibilities were measured at 25°C by the Gouy method using mercuric tetrathiocyanatocobaltate(II) as the magnetic susceptibility standard. Diamagnetic corrections were estimated from Pascal's constant [24]. The magnetic moments were calculated from the equation

(1) $$\begin{array}{c} \mu_{\text{eff.}}=2.84\sqrt{\chi_{M}^{\text{corr}}\cdot T}. \end{array}$$

The molar conductance of 10^−3^ M solution of the complexes in DMSO was measured at 25°C with a Bibby conductometer type MCl. The resistance measured in ohms and the molar conductivities were calculated according to the equation

(2) $$\begin{array}{c} \Lambda_{M}=V*K*\frac{g}{M_{w}}*\Omega , \end{array}$$

where Λ_*M*_ = molar conductivity (Ω^−1^ cm^2^ mol^−1^), *V* = volume of the complex solution (mL), *K* = cell constant (0.92/cm^−1^), *M*_*w*_ = molecular weight of the complex, *g* = weight of the complex (g), Ω = resistance (Ω). ^1^H-NMR spectra of the ligand, and its Zn(II), Cd(II), and Hg(II) complexes were obtained on Perkin-Elmer R32-90-MHz spectrophotometer. Chemical shifts (ppm) were reported relative to TMS. ESR measurements of solid complexes at room temperature were made using a Varian E-109 spectrophotometer, using DPPH as a standard material. Mass spectra were recorded using JEULJMS-AX-500 mass spectrometer.

### 2.2. Preparation of the Ligand and Its Metal Complexes

#### 2.2.1. Preparation of the Ligand [H_2_L]** (1)**

**Figure 1:**
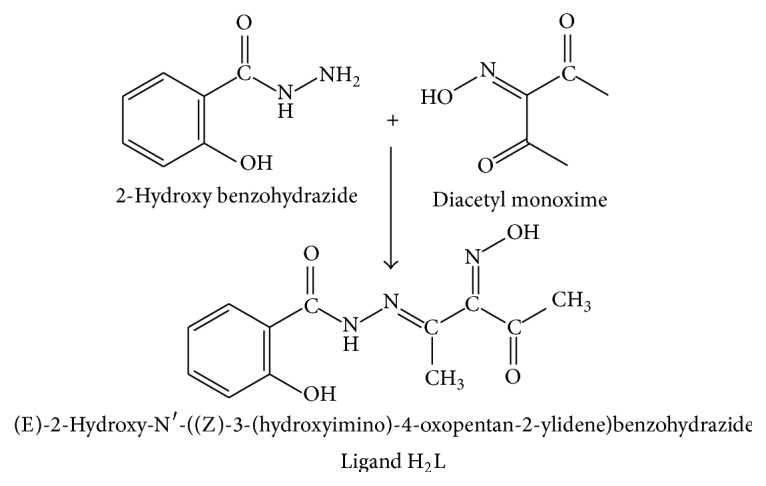
Preparation of the ligand [H_2_L].

The ligand (H_2_L) was prepared by dropwise addition of equimolar amounts of salicylic hydrazide (2-hydroxy-benzohydrazide) (1.52 g, 0.01 mol) dissolved in 20 mL of absolute ethanol to an ethanolic solution of diacetyl monoxime (1.29 g, 0.01 mol) (Figure 1). The mixture was refluxed with stirring for 4 hrs. A dark green precipitate was obtained, filtered off, washed with ethanol, and dried under vacuum over P_2_O_5_. Analytical data of the ligand are given in (Table 1).

#### 2.2.2. Preparation of Metal Complexes,** (2)**–**(17)**

Complexes** (2)**–**(17)** were synthesized by refluxing 25 mL ethanoic solution of the ligand with 25 mL ethanolic solution of 3.79 g, 0.02 mol of Cu(OAc)_2_·H_2_O (1 L : 1 M), complex** (2)**; 7.58 g, 0.04 mol of Cu(OAc)_2_·H_2_O (1 L : 2 M), complex** (3)**; 1.89 g, 0.01 mol of Cu(OAc)_2_·H_2_O (2 L : 1 M), complex** (4)**; 1.88 g, 0.02 mol of CuCl_2_·2H_2_O (1 L : 1 M), complex** (5)**; 3.03 g, 0.02 mol of CuSO_4_·5H_2_O (1 L : 1 M), complex** (6)**; 4.73 g, 0.02 mol of Ni(OAc)_2_·4H_2_O (1 L : 1 M), complex** (7)**; 4.99 g, 0.02 mol of NiSO_4_·6H_2_O (1 L : 1 M), complex** (8)**; 4.73 g, 0.02 mol of Co(OAc)_2_·4H_2_O (1 L : 1 M), complex** (9)**; 2.94 g, 0.02 mol of CoSO_4_·4H_2_O (1 L : 1 M), complex** (10)**; 5.86 g, 0.02 mol of 4.65 g, 0.02 mol of Mn(OAc)_2_·4H_2_O (1 L : 1 M), complex** (11)**; 4.17 g, 0.02 mol of Zn(OAc)_2_·2H_2_O (1 L : 1 M), complex** (12)**; 3.41 g, 0.02 mol of ZnSO_4_·4H_2_O (1 L : 1 M), complex** (13)**; 5.06 g, 0.02 mol of Cd(OAc)_2_·2H_2_O (1 L : 1 M), complex** (14)**; 4.87 g, 0.02 mol of CdSO_4_·8H_2_O (1 L : 1 M), complex** (15)**; 6.05 g, 0.02 mol of Hg(OAc)_2_·4H_2_O (1 L : 1 M), complex** (16)**; 5.13 g, 0.02 mol of FeCl_3_·6H_2_O (1 L : 1 M), complex** (17)**. The reaction mixtures were refluxed with stirring for 2–4 hrs range, depending on the nature of the metal ion and the anion. The precipitates so formed were filtrated off, washed with ethanol, and dried in vacuum over P_2_O_5_. Analytical data of the metal complexes are given in (Table 1).

### 2.3. Biological Activity

Evaluation of the cytotoxic activity of the ligand and its metal complexes was carried out in the Pathology Department, Faculty of Medicine, El-Menoufia University, Egypt. The evaluation process was carried out in vitro using the Sulfo-Rhodamine-B-stain (SRB) [25]. Cells were plated in 96-multiwell plate (10^4^ cells/well) for 24 hrs before treatment with the complexes to allow attachment of cell to the wall of the plate. Different concentrations of the compounds under test in DMSO (0, 5, 12.5, 25, and 50 *μ*g/mL) were added to the cell monolayer, triplicate wells being prepared for each individual dose. Monolayer cells were incubated with the complexes for 48 hrs at 37°C and in atmosphere of 5% CO_2_. After 48 hrs, cells were fixed, washed, and stained with Sulfo-Rhodamine-B-stain. Excess stain was wash with acetic acid and attached stain was recovered with Tris EDTA buffer. Color intensity was measured in an ELISA reader. The relation between surviving fraction and drug concentration is plotted to get the survival curve for each tumor cell line after addition the specified compound.

## 3. Results and Discussion

**Figure 2:**
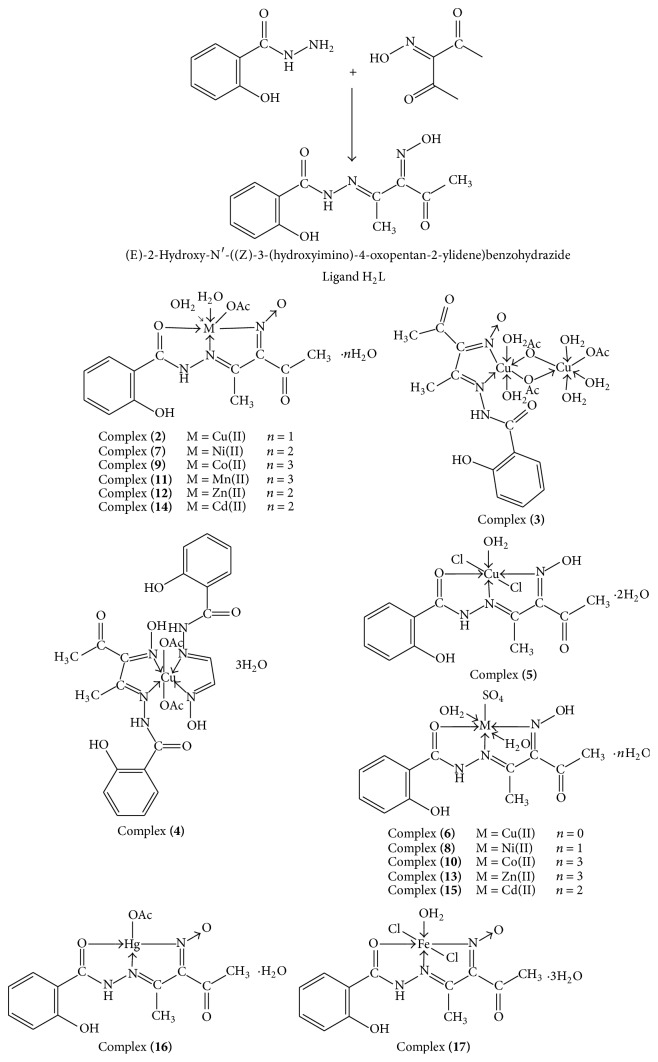
Proposed structures of the ligand [H_2_L] and its metal complexes.

All complexes are colored, stable at room temperature, nonhygroscopic, partially soluble in common organic solvents such as CHCl_3_, and appreciably soluble in DMF and DMSO. The analytical and physical data (Table 1) and spectral data (Tables 2–4) are compatible with the proposed structures (Figure 2). Many attempts have been made to grow up single crystal but no diffractable crystals have been grown till now. The molar conductances of the complexes in 10^−3^ M DMF at 25°C are in the 13.0–33.4 ohm^−1^ cm^2^ mol^−1^ range, indicating a nonelectrolytic nature [26, 27]. The relative high values for some complexes suggest partial dissociation in DMF. The elemental analyses indicated that, all complexes were formed in 1 L : 1 M molar ratio, except complex** (3)** which is found to be formed in 1 L : 2 M molar ratio.

### 3.1. Mass Spectra of the Ligand

The mass spectrum of the [H_2_L], ligand showed the molecular ion peak at* m/e* 263 amu, confirming its formula weight (F.W. 363). The mass fragmentation patterns observed at* m/z* = 76, 93, 109, 121, 123, 137, 180, 215, and 263 amu correspond to C_6_H_4_, C_6_H_4_OH, C_6_H_5_O_2_, C_7_H_5_O_2_, C_7_H_7_O_2_, C_7_H_7_NO_2_, C_8_H_8_N_2_O_3_, C_8_H_13_N_3_O_4_, and C_12_H_13_N_3_O_4_ moieties, respectively, supported the suggested structure of the ligand.

### 3.2. ^1^H-NMR Spectra

The ^1^H-NMR spectrum of the ligand indicated the presence of two peaks at *δ* = 11.83 and 12.07 ppm assigned to proton of anti- and syn-oxime NOH protons, respectively; these two bands disappeared in the presence of D_2_O, indicating that these protons are acidic and the hydroxyl group can participate in the coordination with the metal ions. The syn : anti ratio was found to be 1 : 1, indicating that the percentages of free and hydrogen bonded OHs are identical [28, 29]. Signals at *δ* = 9.73 and *δ* = 10.90 ppm were assigned to the NH protons [28, 30]. The spectrum showed a set of peaks as multiples in the (7.94–6.81 ppm) range, which were assigned to aromatic protons ring [31]. Peaks which appeared at 2.50 and 2.21 ppm were assigned to acetyl and methyl groups respectively [17, 32]. These signals disappeared upon adding D_2_O.

Zn(II), Cd(II), and Hg(II) complexes** (12)**,** (14)**, and** (16)** showed similar spectra. The peaks assigned to the oxime protons disappeared, indicating its participation in the metal coordination. A set of multiple peaks corresponding to the aromatic protons were observed in the 6.88–7.50 ppm range. Signals corresponding to acetyl and methyl prortons appeared at 2.50 and 1.91 ppm, respectively [17, 32]; these signals were disappeared upon adding D_2_O. A new signal was observed around 1.87 ppm, which may be assigned to protons of the coordinated acetate group [33].

### 3.3. IR Spectra

The characteristic infrared spectral data of ligand H_2_L** (1)** and its metal complexes are listed in Table 2. The spectrum of the ligand showed characteristics absorption broad bands in 3360–3315 cm^−1^ range, which are due to intra- and intermolecular hydrogen bonding of OH of the oxime groups with the imino nitrogen and carbonyl oxygen atoms [22, 30]. The medium band at 3215 cm^−1^ was assigned to (NH) group [29]. The band appearing at 1700 cm^−1^ was assigned to *υ*(C=O) band, which is less than the expected value. It is deduced, therefore, that the carbonyl group is involved in hydrogen bondings in the ligand. On the other hand, the *υ*(C=O) band of the amide group appeared at 1664 cm^−1^ [29]. The *υ*(C=N) vibrations (imine and oxime groups) appeared at 1613 and 1587 cm^−1^, respectively [29, 32]. Two strong bands observed at 1147 and 1000 cm^−1^ which were assigned to *υ*(N–O) [22, 34]. The splitting of the *υ*(N–O) vibration into two bands confirmed the presence of two nonequivalent hydrogen bonding formations whereby the intramolecular type is stronger than the intermolecular type. The bonding mode of the ligand in the metal complexes has been deduced by comparing the IR spectra of the complexes with that of the free ligand. Ir spectra showed that the ligand coordinated through the nitrogen atoms of the imine and the oximato (C=N→O) groups. This mode of bonding is supported by negative shifts in bands of these groups and simultaneous increasing in (N→O) band, appearing in the 1150–1170 cm^−1^ range [35, 36]. In all complexes except complexes** (3)** and** (4)**, the strong band assigned to *υ*(C=O) amid stretching band was shifted to lower frequency, indicating involvement of amide keto oxygen in the metal coordinating. The appearance of two characteristic bands at 1482, 1483, 1500, and 1345, 1382, 1376 cm^−1^ in spectra of complexes** (3)**,** (4)**, and** (16)**, respectively, were attributed to *υ*_asym._(COO^−^) and *υ*_sym._(COO^−^), respectively, indicating the participation of the acetate oxygen in the complex formation [37]. The mode of coordination of acetate group has often been deduced from the magnitude of the observed separation between the *υ*_asym._(COO^−^) and *υ*_sym._(COO^−^). The separation value (Δ) between *υ*_asym._(COO^−^) and *υ*_sym._(COO^−^) for these complexes were 137, 101, and 124 cm^−1^ suggesting the coordination of acetate group in a monodentate fashion [38, 39]. In addition, complex** (3)** showed *υ*(CO_2_) at 1560 and 1425 cm^−1^ due to a bridging acetate group. The chloro complexes** (5)** and** (17)** showed new bands at 443 and 482 cm^−1^, respectively, this band was assigned to *ν*(M–Cl), whereas sulphate complexes** (6)**,** (8)**,** (10)**,** (13)**, and** (15)** exhibited new bands in the (1232–1260), (1004–1115), (850–885), and (650–690) cm^−1^ ranges, these values indicated that the sulphate ion is coordinated to the metal ion in a unidentate chelating fashion [35, 40]. The mode of coordination is supported by presence of additional bands in 620–528 and 682–592 cm^−1^ regions corresponding to *υ*(M–N) and *υ*(M–O) bands, respectively [22, 36, 41].

### 3.4. Electronic Spectra and Magnetic Moments

The electronic absorption spectral data of the ligand and its metal complexes in DMF are listed in Table 3. The ligand showed three bands at 275 nm (5.86 × 10^−4^ L mol^−1^ cm^−1^) and 315 nm (ɛ = 8.45 × 10^−4^ L mol^−1^ cm^−1^) and 350 nm (ɛ = 9.2 × 10^−4^ L mol^−1 ^cm^−1^). The first band may be assigned to ***π*** → ***π*** ^*∗*^ transition which is nearly unchanged upon complexation, whereas the second and third bands may be assigned to the **n** → ***π*** ^*∗*^ and charge transfer transitions of the azomethine and carbonyl groups [30, 42]. These two bands were shifted to lower energy upon complex formation, indicating participation of these groups in coordination with the metal ions. The electronic spectra of copper(II) complexes** (2)**–**(6)** were nearly identical and showing bands centered in the 425–505, 550–595, and 605–626 nm ranges assigned to ^2^B_1g_ → ^2^A_1g_ *υ*_1_(d_*x*^2^-*y*^2^_ → d_*z*^2^_),  ^2^B_1g_ → ^2^B_2g_, *υ*_2_(d_*x*^2^-*y*^2^_ → d_*xy*_), and ^2^B_1g_ → ^2^E_g_, *υ*_3_(d_*x*^2^-*y*^2^_ → d_*xy*_, d_*yz*_) transitions, respectively. These transitions indicated that the copper(II) ion has a tetragonally distorted octahedral geometry. This could be due to the Jahn-Teller effect that operates on the d^9^ electronic ground state of six coordinate system, elongating one trans pair of coordinate bonds and shortening the remaining four ones [43]. The magnetic moments for copper(II) complexes at room temperature were in the 1.68–1.81 range BM, supporting that the complexes have octahedral geometry [44]. The low magnetic moments values of complexes** (3)** and** (4)** may be due spin-spin interactions between copper(II) [44]. Nickel(II) complexes** (7)** and** (8)** displayed three bands at 420, 617, 749 and 415, 620, 720 nm, respectively, these bands were assigned to ^3^A_2g_(F) → ^3^T_2g_(F)  *υ*_1_, ^3^A_2g_(F) → ^3^T_2g_  (*υ*_2_), and ^3^A_2g_(F) → ^3^T_1g_(P)  *υ*_3_ transitions, indicating octahedral nickel(II) complexes [26, 45]. The *υ*_2_/*υ*_1_ ratios were 1.06 and 1.04 for complexes** (7)** and** (8)**, respectively, which are less than the usual range of 1.5–1.75, indicating distorted octahedral nickel(II) complex [26, 45]. The values of magnetic moments for nickel(II) complex** (7)** and** (8)** were 2.82 and 2.85 BM, respectively, which are consistent with two unpaired electrons state, confirming octahedral geometry for nickel(II) [45]. The cobalt(II) complexes** (9)** and** (10)** exhibited only two bands at (598, 620) and (556, 621) nm ranges, respectively. These bands were assigned to ^4^T_1g_(F) → ^4^T_2g_(F)  *υ*_1_ and ^4^T_1g_(F) → ^4^A_2g_(F)  *υ*_2_ transitions, respectively. The third transition band has not been observed possibly because it is out the range of spectrophotometer (>900 nm). The lower value of *υ*_2_/*υ*_1_ complex** (9)** and complex** (10)** may be due to distortion of the octahedral structures [26]. The magnetic moments for cobalt(II) complexes** (9)** and** (10)** at room temperature recorded 5.11 and 5.52 BM, respectively. These values are consistent with high spin cobalt(II) ion (d^7^). Manganese(II) complex** (11)** displayed weak bands at 425, 585, and 611 nm. These bands were assigned to ^6^A_1g_ → ^4^E_g_, ^6^A_1g_ → ^4^T_2g_, and ^6^A_1g_ → ^4^T_1g_ transitions, respectively, corresponding to an octahedral structure for manganese(II) complex [43, 46]. Since all the excited states of Mn(II) ion either quartets or doublets, the absorption spectra of Mn(II) ions have only spin forbidden transitions. Therefore, the intensity of transitions was weak. The value of magnetic moment for manganese(II) complex** (11)** is 6.10 BM which is consistent with high spin octahedral geometry for manganese(II) [41, 47]. Iron(III) complex** (17)** showed bands at 475, 541, and 615 nm. The first transition is related to charge transfer from the ligand to iron(III) ion, whereas the other two bands were assigned to ^6^A_1g_ → ^4^T_1g_ and ^6^A_1g_ → ^4^T_2g_ transitions, suggesting a distorted octahedral structure around the iron atom [46, 48]. The recorded magnetic moment (6.11 BM) is consistent with the proposed high spin octahedral geometry for iron(III) complex [41, 47]. The observed bands in zinc(II)** (12)** and** (13)**, cadmium(II)** (14)** and** (15)**, and mercury(II)** (16)** complexes (Table 3) are due to interligand transitions within the ligand.

### 3.5. Electron Spin Resonance (ESR)

The ESR spectra of solid copper(II) complexes** (2)**–**(5)** at room temperature are characteristic of a species with a d^9^ configuration and having an axial symmetry type of a d_(*x*^2^-*y*^2^)_ ground state, which is the most common for copper(II) complexes [22]. Complexes** (2)** and** (3)** showed isotropic type with *g*_iso_ = 2.18 and 2.10, whereas complexes** (4)** and** (5)** showed axial type with *g*_||_ > *g*_⊥_ > 2.04, indicating a tetragonal distortion [26, 33], corresponding to elongation along the fold symmetry axis *Z*. The *g*-values are related by the expression *G* = (*g*_||_ − 2)/(*g*_⊥_ − 2). If *G* > 4.0, then, the local tetragonal axes are aligned parallel or only slightly misaligned; if *G* < 4.0, the significant exchange coupling is present. Complexes** (4)** and** (5)** showed *G* value ≥ 4.0, indicating that tetragonal axes are present. Also, these complexes showed g_||_ ≤ 2.3, suggesting considerable covalent bond character around the copper(II) ion [49, 50]. Also, the in-plane *σ*-covalence parameter, *α* ^2^(Cu), was calculated by

(3) α2Cu=A||0.036+g||−2.002+37g⊥−2.002+0.04.

The calculated values for** (4)** and** (5)** are 0.76 and 0.67 (Table 4), suggesting covalent bond character [26, 51]. The *g*_||_/*A*_||_ is taken as an indication for the stereochemistry of the copper(II) complexes. Karlin has suggested that this ratio may be an empirical indication of the stereochemistry of copper(II) complex [52]. The value *g*_||_/*A*_||_ quotient in the (165–173.1) cm^−1^ range is expected for copper(II) complexes within perfectly square based geometry and those higher than 150 cm^−1^ for tetragonally distorted complexes. The values for copper(II) complexes** (4)** and** (5)** are associated with a tetragonally distorted field around copper(II) centers. For copper(II) complexes with ^2^B_1_ ground state, the *g*-values can be related to the parallel (*K*_||_) and perpendicular (*K*_⊥_) components of the orbital reduction factor (*K*_⊥_) as follows [51]:

(4) K||2=g||−2.0023ΔExy8λo,K⊥2=g⊥−2.0023ΔExz2λo,K2=K||2+2K⊥23,

where *λ*_*o*_ is the spine orbit coupling of free copper ion (−828 cm^−1^) and Δ*E*_*xy*_ and Δ*E*_*xz*_ are the electronic transition energies of ^2^B_1_→^2^B_2_ and ^2^B_1_→^2^E, respectively. For the purpose of calculation, it was assumed that the maximum in the band corresponds to Δ*E*_*xy*_ and Δ*E*_*xz*_ can be taken from the wavelength of these bands. From the above relations, the orbital reduction factors (*K*_||_, *K*_⊥_, and *K*) which are a measure of covalence can be calculated. For an ionic environment, *K* = 1, and for a covalent environment, *K* < 1, the lower the value of *K*, the greater the covalent character. The values of *K* for** (4)** and** (5)** (Table 4) showed considerable covalent bond character. The in-plane and out-of-plane *π*-bonding coefficients (*β*_1_^2^ and *β* ^2^), respectively, are dependent upon to values of Δ*E*_*xy*_ and Δ*E*_*xz*_ in the following equations [53]:

(5) α2β2=g⊥−2.002ΔE2λο,α2β12=g||−2.002ΔE8λο.

Complexes** (4)** and** (5)** showed *β*_1_^2^ values 1.1 and 1.0, indicating a moderate degree of ionic character in the in-plane *π*-bonding, while *β* ^2^ are 0.92 and 0.94, indicating ionic character in the out-of-plane *π*-bonding [54]. It is possible to calculate the approximate orbital population for d orbital using the following equations [22]:

(6) A||=Aiso−2B1±74Δg||,ad2=2B2Bo,

where 2*B* ^*o*^ is the calculated dipolar coupling for unit occupancy of the d orbital. When the data of complexes** (4)** and** (5)** are analyzed, the results suggested an orbital population close to 72.4 and 77.7% d-orbital spin density clearly, the orbit of the unpaired electron is a d_(*x*^2^-*y*^2^)_ ground state [22]. The ESR spectral data, for copper(II) complexes are shown in Table 4. Co(II)** (9)** and Mn(II)** (11)** complex showed isotropic spectra with *g*_iso_ = 2.1 and 2.003, respectively.

### 3.6. Thermal Analyses (DTA and TGA)

IR spectral data (Table 2) indicates the presence of water molecules; thermal analyses were carried out to ascertain their nature, and to give an insight into the thermal stability of the studied compounds. The results showed that there is a good agreement in the weight loss between the calculated and the proposed formulae. The thermal analyses imply that all complexes generally decomposed in several steps (Table 5). The DTA and TGA thermogram of complex** (4)** showed that the complex decomposed in four steps. The first peak at 120°C with a weight loss of 7.0% (calcd. 7.08%) is assigned to elimination of three hydrated water molecules, which is accompanied by an endothermic peak. The second step appeared as an exothermic peak at 160°C, assigned to loss of two CH_3_COOH molecules with weight loss 7.81% (calcd. 7.74%). The third step appeared as an exothermic peak at 298°C, referring to melting point of the complex. The fourth step at 475°C with a weight loss of 73.36% (calcd. 74.48) implies completing decomposition of this complex that ended with the formation CuO that is accompanied by an exothermic peak. The TG and DTA thermogram of complex** (8)** showed that the complex decomposed in four steps. The first peak at 90°C with a weight loss of 3.69% (calcd. 3.60%) assigned to the elimination one hydrated water molecule, which is accompanied by an endothermic peak. The second step at 195°C with a weight loss of 7.48% (calcd. 7.39%) is assigned to elimination of two coordinating water molecules that is accompanied by an endothermic peak. The third step appeared as an exothermic peak at 275°C, assigned to loss of one H_2_SO_4_ molecule with weight loss 20.27 (calcd. 20.13%). The fourth step appeared as an exothermic peak at 400°C, referring to melting point of the complex. The fifth step at 460°C with a weight loss of 67.51% (calcd. 68.73) implies the complete decomposition of this complex that ended with the formation NiO that is accompanied by an exothermic peak. The TG and DTA thermogram of complexes** (9)** and** (11)** showed that these complexes decomposed in five steps. The first peak appeared at 79 and 90°C with a weight loss of 11.60 and 11.87% (calcd. 11.49 and 11.74%), respectively, is assigned to removal of three hydrated water molecules from each complex; this weight loss is accompanied by an endothermic peak. The second peak at 140 and 170°C with a weight loss of 7.24 and 7.90% (calcd. 7.35 and 7.73%) is assigned to elimination of two coordinated water molecules from each complex; this is accompanied by an endothermic peak. The third step at 170 and 258 associated a weight loss of 12.46 and 12.86% (calcd. 12.25 and 13.04%) is assigned to the elimination of one acetate ion from each complex, this step accompanied with endothermic peak. The fourth step appeared as an exothermic peak at 325 and 319°C, referring to the melting point of the two complexes, respectively. The fifth step at 450 and 445°C with a weight loss of 51.79 and 64.13% (calcd. 52.98 and 65.18) implies to complete decomposition of these complexes, respectively, leaving metal oxide that is accompanied by an exothermic peak:

(7) HLCuOAcH2O2·2H2O→60°CHLCuOAcH2O2·2H2OHLCuOAcH2O2→150°CHLCuOAc+2H2OHLCuOAc→265°CLCu+CH3COOHLCu→570°CCuO+organic  residues

### 3.7. Cytotoxicity Activity

**Figure 3:**
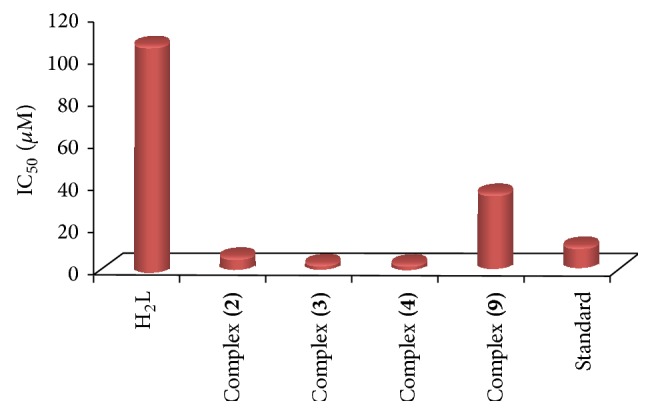
IC_50_ values of the ligand H_2_L** (1)** and its metal complexes** (2)**,** (3)**,** (4)**, and** (9)** against human liver HepG2 cancer cell.

The cytotoxic activity of the oxime hydrazone ligand H_2_L** (1)** and its metal complexes** (2)**,** (3)**,** (4)**, and** (9)** was evaluated against human liver HepG2 cancer cell, (HepG2 cell line) within 0.1–100 *μ*g/L concentration range. The IC_50_ values were calculated for each compound and results are presented in Figure 3 and Table 6. As shown, most complexes displayed significantly cytotoxic activities compared to Sorafenib (Nexavar) standard drug. It seems that changing the anion, coordination sites, and the nature of the metal ion has effect on the biological behavior. Cytotoxicity activity of the complexes may be attributed to the central metal atom which was explained by Tweedy's chelation theory [55, 56]. Cytotoxicity results indicated that all tested complexes (IC_50_ = 2.24–6.49 *μ*M) (except complex** (9)** with IC_50_ = 36.80 *μ*M) demonstrated potent cytotoxicity against HepG2 cancer cells. Copper complex** (4)** showed the highest cytotoxicity effect with IC_50_ value of 2.24 *μ*M, followed by complex** (3)** with IC_50_ value 2.67 *μ*M and then complex** (2)** with IC_50_ value 6.49 *μ*M. It was observed also that all complexes are more active than the free ligand. This indicated enhancing of the antitumor activity upon coordination. The enhancement of cytotoxic activity may be assigned to that the positive charge of the metal increased the acidity of coordinated ligand that bears protons, leading to stronger hydrogen bonds which enhanced the biological activity [57, 58]. It seems that changing the anion, coordination sites, and the nature of the metal ion has a pronounced effect on the biological behavior by altering the binding ability of DNA [59, 60]. Gaetke and Chow had reported that metal has been suggested to facilitate oxidative tissue injury through a free-radical mediated pathway analogous to the Fenton reaction [61].

## 4. Conclusions

New copper(II), Nickel, cobalt(II), manganese(II), zinc(II) and cadmium(II), Mercury(II), and iron(II) metal complexes derived from N′-(3-(hydroxyimino)-4-oxopentan-2-ylidene) salicylic hydrazide (H_2_L) were synthesized. The analytical and physicochemical data confirmed the composition and structure of the newly obtained compounds. The ligand behaved as monobasic tridentate, monobasic bidentate, neutral tetradentate, and neutral tridentate. The complexes adopted distorted octahedral geometry around the metal ion. The ligand and tested complexes showed a high potential cytotoxic activity against growth human liver cancer HepG2 tumor cell lines) compared to Sorafenib (Nexavar) standard drug. All complexes were found to be more active than the free ligand. This indicates enhancing of antitumor activity upon coordination. Cupper complex** (4)** showed the highest cytotoxic activity with IC_50_ 2.24 *μ*M followed by complex 2.67 *μ*M. These compounds are promising candidates as anticancer agents because of their high cytotoxic activity.

## Figures and Tables

**Table 1:** Analytical and physical data of the ligand [H_2_L] **(1)** and its metal complexes.

Comp. number	Molecular formula	Color	FW	M.P (°C)	Yield (%)	Anal./found (calc.) (%)					Molar conductance^*∗*^
						C	H	N	M	Cl	
**(1)**	[H_2_L] C_12_H_13_N_3_O_4_	Dark green	263	120	80	55.01 (54.75)	5.48 (4.98)	16.01 (15.96)	—	—	

**(2)**	[(HL)Cu(OAc)(H_2_O)_2_]·H_2_O C_14_H_21_N_3_O_9_Cu	Green	439	>300	85	39.02 (38.31)	4.61 (4.82)	9.33 (9.57)	14.50 (14.48)	—	10.12

**(3)**	[(HL)Cu_2_(OAc)_3_(H_2_O)_5_]·H_2_O C_18_H_33_N_3_O_15_Cu_2_	Greenish brown	658.56	>300	90	33.00 (32.83)	5.11 (5.05)	6.51 (6.38)	19.81 (19.30)	—	13.15

**(4)**	[(H_2_L)_2_Cu(OAc)_2_]·3H_2_O C_28_H_38_N_6_O_15_Cu	Yellowish green	762	>300	90	44.47 (44.12)	4.39 (5.03)	11.22 (11.03)	8.52 (8.34)	—	11.30

**(5)**	[(H_2_L)CuCl_2_(H_2_O)]·2H_2_O C_12_H_19_Cl_2_N_3_O_7_Cu	Brownish green	452	>300	85	32.10 (31.90)	4.23 (4.24)	9.52 (9.30)	14.30 (14.07)	16.00 (15.70)	14.10

**(6)**	[(H_2_L)Cu(SO_4_)(H_2_O)_2_] C_12_H_17_N_3_O_10_SCu	Dark olive	459	>300	80	31.71 (31.41)	4.00 (3.73)	9.72 (9.16)	14.10 (13.85)	—	16.20

**(7)**	[(HL)Ni(OAc)(H_2_O)_2_]·2H_2_O C_14_H_23_N_3_O_10_Ni	Pale brown	452	>300	70	37.59 (37.20)	4.71 (5.13)	9.82 (9.30)	13.21 (12.98)	—	10.87

**(8)**	[(H_2_L)Ni(SO_4_)(H_2_O)_2_]·H_2_O C_13_H_22_N_3_O_11_SNi	Yellow	487	>300	90	32.32 (32.06)	4.81 (4.55)	9.10 (8.63)	12.52 (12.05)	—	15.70

**(9)**	[(HL)Co(OAc)(H_2_O)_2_]·3H_2_O C_14_H_25_N_3_O_11_CO	Dark brown	470	>300	78	35.98 (35.75)	5.51 (5.36)	9.12 (8.93)	13.00 (12.53)	—	11.81

**(10)**	[(H_2_L)Co(SO_4_)(H_2_O)_2_]·3H_2_O C_13_H_26_N_3_O_13_SCo	Pale white	523	>300	70	30.12 (29.83)	5.31 (5.01)	8.52 (8.03)	11.82 (11.26)	—	15.87

**(11)**	[(HL)Mn(OAc)(H_2_O)_2_]·3H_2_O C_14_H_25_N_3_O_11_Mn	Brown	466	>300	75	35.66 (36.06)	5.12 (5.40)	9.33 (9.01)	12.12 (11.78)	—	11.12

**(12)**	[(HL)Zn(OAc)(H_2_O)_2_]·2H_2_O C_14_H_23_N_3_O_10_Zn	Brown green	459	>300	75	37.00 (36.65)	5.51 (5.05)	9.72 (9.16)	14.73 (14.25)	—	11.87

**(13)**	[(H_2_L)Zn(SO_4_)(H_2_O)_2_]·3H_2_O C_12_H_23_N_3_O_13_SZn	Reddish brown	515	>300	70	28.33 (28.01)	4.62 (4.500)	8.51 (8.16)	13.11 (12.70)	—	15.72

**(14)**	[(HL)Cd(OAc)(H_2_O)_2_]·2H_2_O C_14_H_23_N_3_O_10_Cd	White	506	>300	80	33.72 (33.25)	4.72 (4.58)	8.71 (8.31)	22.87 (22.23)	—	12.11

**(15)**	[(H_2_L)Cd(SO_4_)(H_2_O)_2_]·2H_2_O C_12_H_21_N_3_O_12_SCd	Yellowish white	544	>300	85	27.01 (26.50)	3.95 (3.89)	8.11 (7.73)	21.10 (20.67)	—	17.11

**(16)**	[(HL)Hg(OAc)]·H_2_O C_14_H_17_N_3_O_7_Hg	Yellowish green	540	>300	70	31.52 (31.15)	3.32 (3.17)	8.11 (7.78)	37.52 (37.15)	—	9.82

**(17)**	[(HL)Fe(Cl)_2_(H_2_O)]·3H_2_O C_12_H_20_N_3_O_8_Cl_2_Fe	Black	461	>300	75	31.65 (31.26)	4.72 (4.37)	9.35 (9.11)	12.45 (12.11)	15.87 (15.38)	14.87

^*∗*^Λm (Ω^−1^ cm^2^ mol^−1^).

**Table 2:** IR frequencies of the bands (cm^−1^) of ligand [H_2_L], **(1)** and its metal complexes.

Comp. number	Molecular formula	*ν*(H_2_O/OH)	*ν*(NH)	*ν*(C=O)_acetyl_	*ν*(C=O)_amide_	*ν*(C=N)_imine_	*ν*(C=N)_oxime_	*ν*(N–O)	*ν*(OAc)/SO_4_	*ν*(M–O)	*ν*(M–N)	*ν*(M–Cl)
**(1)**	[H_2_L]C_12_H_13_N_3_O_4_	3360, 3315	3215	1700	1664	1613	1587	1147, 1000, 931	—	—	—	—

**(2)**	[(HL)Cu(OAc)(H_2_O)_2_]·H_2_O C_14_H_21_N_3_O_9_Cu	3430 3520–3080	3260	1680	1650	1605	1575	1155, 1040, 925	1443, 1342	600	575	—

**(3)**	[(HL)Cu_2_(OAc)_3_(H_2_O)_5_]· C_18_H_33_N_3_O_15_Cu_2_	3431 3575–3000	3260	1709	1652	1601	1567	1170, 1066 930	1482, 1345	682	582	—

**(4)**	[(H_2_L)_2_Cu(OAc)_2_]·3H_2_O C_28_H_38_N_6_O_15_Cu	3421 3500–3180	3217	1712	1669	1601	1564	1163, 1068 925	1483, 1382	679	580	—

**(5)**	[(H_2_L)CuCl_2_(H_2_O)]·2H_2_O C_12_H_19_Cl_2_N_3_O_7_Cu	3434–3380	3141	1695	1659	1606	1533	1154, 1128 1013, 954	—	662	575	443

**(6)**	[(H_2_L)Cu(SO_4_)(H_2_O)_2_] C_12_H_17_N_3_O_10_SCu	3500 3250–3100	3202	1717	1667	1611	1529	1190, 1104 977, 915	1260, 1071, 867, 650	618	522	—

**(7)**	[(HL)Ni(OAc)(H_2_O)_2_]·2H_2_O C_14_H_23_N_3_O_10_Ni	3464 3522–3247	3217	1760	1674	1605	1564	1150 1024, 909	1456, 1339	682	620	—

**(8)**	[(H_2_L)Ni(SO_4_)(H_2_O)_2_]·H_2_O C_13_H_22_N_3_O_11_SNi	3428 3560–3280	3165	1700	1670	1609	1531	1160, 1102 1043, 925	1240, 1102, 850, 690	592	528	—

**(9)**	[(HL)Co(OAc)(H_2_O)_2_]·3H_2_O C_14_H_25_N_3_O_11_CO	3495 3560–3300	3189	1717	1671	1609	1526	1150, 1065 1022, 920	1465, 1326	624	535	

**(10)**	[(H_2_L)Co(SO_4_)(H_2_O)_2_]·3H_2_O C_13_H_26_N_3_O_13_SCo	3300 3560–3215	3215	1700	1652	1610	1553	1152, 1030, 956	1232, 1092 858, 650	605	545	—

**(11)**	[(HL)Mn(OAc)(H_2_O)_2_]·3H_2_O C_14_H_25_N_3_O_11_Mn	3408 3570–3300	3217	1695	1600	1595	1565	1170, 1149 1029, 920	1460, 1338	654	587	

**(12)**	[(HL)Zn(OAc)(H_2_O)_2_]·2H_2_O C_14_H_23_N_3_O_10_Zn	3315 3600–3280	3214	1700	1673	1601	1566	1154, 1039, 985	1523, 1391	597	535	

**(13)**	[(H_2_L)Zn(SO_4_)(H_2_O)_2_]·3H_2_O C_12_H_23_N_3_O_13_SZn	3380 3580–3280	3285	1717	1653	1603	1552	1154, 1042, 951	1501, 1366	589	537	

**(14)**	[(HL)Cd(OAc)(H_2_O)_2_]·2H_2_O C_14_H_23_N_3_O_10_Cd	3380 3587–3210	3206	1705	1675	1613	1534	1155, 1044, 979	1237, 1115, 1060, 885, 650	616	526	

**(15)**	[(H_2_L)Cd(SO_4_)(H_2_O)_2_]·2H_2_O C_12_H_21_N_3_O_12_SCd	3428 3652–3275	3217	1710	1652	1587	1565	1160, 1014, 956	1500, 1376	612	588	

**(16)**	[(HL)Hg(OAc)]·H_2_O C_14_H_17_N_3_O_7_Hg	3365 3525–3260	3200	1710	1644	1609	1543	1151, 1008, 936	—	665	524	482

**(17)**	[(HL)Fe(Cl)_2_(H_2_O)]·3H_2_O C_12_H_20_N_3_O_8_Cl_2_Fe											

**Table 3:** Electronic spectra (nm) and magnetic moments (BM) for the ligand **(1)** and metal complexes.

Comp. number	Molecular formula	*λ*_max_ (nm)	*μ*_eff_ (BM)	*ν*_2_/*ν*_1_
**(1)**	[H_2_L]C_12_H_13_N_3_O_4_	275 nm (*ε* = 5.86 × 10^−4^ L mol^−1^ cm^−1^) 315 nm (*ε* = 8.45 × 10^−4^ L mol^−1^ cm^−1^) 350 nm (*ε* = 9.20 × 10^−4^ L mol^−1^ cm^−1^)	—	—

**(2)**	[(HL)Cu(OAc)(H_2_O)_2_]·H_2_O C_14_H_21_N_3_O_9_Cu	265, 302, 380, 495, 550, 625	1.79	—

**(3)**	[(HL)Cu_2_(OAc)_3_(H_2_O)_5_]· C_18_H_33_N_3_O_15_Cu_2_	268, 308, 380, 425, 550, 626	1.68	—

**(4)**	[(H_2_L)_2_Cu(OAc)_2_]·3H_2_O C_28_H_38_N_6_O_15_Cu	270, 370, 390, 450, 570, 610	1.81	—

**(5)**	[(H_2_L)CuCl_2_(H_2_O)]·2H_2_O C_12_H_19_Cl_2_N_3_O_7_Cu	260, 300, 370, 460, 565, 605	1.77	—

**(6)**	[(H_2_L)Cu(SO_4_)(H_2_O)_2_] C_12_H_17_N_3_O_10_SCu	265, 300, 400, 505, 595, 605	1.78	—

**(7)**	[(HL)Ni(OAc)(H_2_O)_2_]·2H_2_O C_14_H_23_N_3_O_10_Ni	272, 303, 420, 525, 617, 687, 749	2.85	1.06

**(8)**	[(H_2_L)Ni(SO_4_)(H_2_O)_2_]·H_2_O C_13_H_22_N_3_O_11_SNi	265, 300, 415, 510, 620, 690, 720	2.95	1.04

**(9)**	[(HL)Co(OAc)(H_2_O)_2_]·3H_2_O C_14_H_25_N_3_O_11_CO	275, 301, 420, 598, 620	5.11	1.03

**(10)**	[(H_2_L)Co(SO_4_)(H_2_O)_2_]·3H_2_O C_13_H_26_N_3_O_13_SCo	302, 397, 430, 556, 621, 265	5.52	1.11

**(11)**	[(HL)Mn(OAc)(H_2_O)_2_]·3H_2_O C_14_H_25_N_3_O_11_Mn	265, 298, 390, 425, 585, 611	6.10	—

**(12)**	[(HL)Zn(OAc)(H_2_O)_2_]·2H_2_O C_14_H_23_N_3_O_10_Zn	270, 305, 365	Diamagnetic	—

**(13)**	[(H_2_L)Zn(SO_4_)(H_2_O)_2_]·3H_2_O C_12_H_23_N_3_O_13_SZn	265, 300, 378	Diamagnetic	—

**(14)**	[(HL)Cd(OAc)(H_2_O)_2_]·2H_2_O C_14_H_23_N_3_O_10_Cd	268, 305, 360	Diamagnetic	—

**(15)**	[(H_2_L)Cd(SO_4_)(H_2_O)_2_]·2H_2_O C_12_H_21_N_3_O_12_SCd	265, 302, 370	Diamagnetic	—

**(16)**	[(HL)Hg(OAc)]·H_2_O C_14_H_17_N_3_O_7_Hg	260, 305, 370	Diamagnetic	—

**(17)**	[(HL)Fe(Cl)_2_(H_2_O)]·3H_2_O C_12_H_20_N_3_O_8_Cl_2_Fe	265, 302, 330, 475, 541, 615	6.11	—

**Table 4:** ESR data for metal(II) complexes **(2)–(5)**.

Complex	*g*_||_	*g*_⊥_	*g*_iso_^a^	*A*_||_ (*G*)	*A*_⊥_ (*G*)	*A*_iso_^b^ (*G*)	*G* ^c^	Δ*E*_*xy*_ (cm^−1^)	Δ*E*_*xz*_ (cm^−1^)	*K*_⊥_^2^	*K*_||_^2^	*K*	*g*_||_/*A*_||_ (cm^−1^)	*α* ^2^	*β* ^2^	*β*_1_^2^	2*β*	*a*_d_^2^ (%)
**(2)**	—	—	2.18	—	—	—	—	—	—	—	—	—	—	—	—	—	—	—
**(3)**	—	—	2.10	—	—	—	—	—	—	—	—	—	—	—	—	—	—	—
**(4)**	2.31	2.06	2.14	135	15	55	5.2	18181	20202	0.7	0.84	0.86	165	0.76	0.92	1.1	170	72.4
**(5)**	2.25	2.05	2.12	120	12	48	5	17857	21739	0.63	0.67	0.8	173.1	0.67	0.94	1	182	77.7

^a^3*g*_iso_ = (*g*_||_ + 2*g*_⊥_).

^b^3*A*_iso_ = (*A*_||_ + 2*A*_⊥_).

^c^ *G* = (*g*_||_ − 2)/(*g*_⊥_ − 2).

**Table 5:** Thermal analyses for metal complexes.

Comp. number	Molecular formula	Temp. (°C)	DTA (peak)		TGA (Wt. loss %)		Assignments
			Endo	Exo	Calc.	Found	
**(4)**	[(H_2_L)_2_Cu(OAc)_2_]·3H_2_O C_28_H_38_N_6_O_15_Cu	120	Endo	—	7.08	7.00	Loss of hydrated 3H_2_O
		160	Endo	—	8.33	8.27	Loss of CH_3_COOH
		240	Endo	—	9.09	9.02	Loss of CH_3_COOH
		298	Endo	—	—	—	Melting point
		350	—	Exo	—	—	
		395	—	Exo	—	—	
		475	—	Exo	13.47	13.53	Decomposition process with the formation of CuO

**(8)**	[(H_2_L)Ni(SO_4_)(H_2_O)_2_]·H_2_O C_13_H_22_N_3_O_11_SNi	92	Endo	—	3.69	3.38	Loss of hydrated 1H_2_O
		186	Endo	—	7.67	7.62	Loss of coordinated 2H_2_O
		275	Endo	—	22.17	22.03	Loss of H_2_SO_4_
		314	Endo	—	—	—	Melting point
		400	—	Exo	—	—	
		420	—	Exo	—	—	
		460	—	Exo	22.16	22.88	Decomposition process with the formation of NiO

**(9)**	[(HL)Co(OAc)(H_2_O)_2_]·3H_2_O C_14_H_25_N_3_O_11_CO	79	Endo	—	11.48	11.01	Loss of hydrated 3H_2_O
		140	Endo	—	8.65	8.47	Loss of coordinated 2H_2_O
		170	Endo	—	15.52	15.25	Loss of CH_3_COOH
		325	Endo	—	—	—	Melting point
		400	—	Exo	—	—	
		430	—	Exo	—	—	
		450	—	Exo	13.39	13.55	Decomposition process with the formation of CoO

**(11)**	[(HL)Mn(OAc)(H_2_O)_2_]·3H_2_O C_14_H_25_N_3_O_11_Mn	90	Endo	—	11.58	11.86	Loss of hydrated 3H_2_O
		170	Endo	—	8.73	8.47	Loss of coordinated 2H_2_O
		258	Endo	—	15.65	15.69	Loss of CH_3_COOH
		319	Endo	—	—	—	Melting point
		390	—	Exo	—	—	
		413	—	Exo	—	—	
		445	—	Exo	12.93	12.71	Decomposition process with the formation of MnO

**Table 6:** Cytotoxic activity (IC_50_) of the ligand and some metal complexes against human liver HepG2 cancer.

Number	Compound	IC_50_ (*μ*M)
Ligand	H_2_L, **(1)**	107 ± 5.2
**(2)**	[(HL)Cu(OAc)(H_2_O)_2_]·H_2_O	6.49 ± 1.7
**(3)**	[(HL)Cu_2_(OAc)_3_(H_2_O)_5_]	2.67 ± 1.2
**(4)**	[(H_2_L)_2_Cu(OAc)_2_]·3H_2_O	2.24 ± 1.2
**(9)**	[(HL)Co(OAc)(H_2_O)_2_]·3H_2_O	36.80 ± 4.0
Standard	Sorafenib (Nexavar)	11.8 ± 3.2
